# Qualitative Evidence Synthesis (QES) for Guidelines: Paper 3 – Using qualitative evidence syntheses to develop implementation considerations and inform implementation processes

**DOI:** 10.1186/s12961-019-0450-1

**Published:** 2019-08-08

**Authors:** Claire Glenton, Simon Lewin, Theresa A. Lawrie, María Barreix, Soo Downe, Kenneth W. Finlayson, Tigest Tamrat, Sarah Rosenbaum, Özge Tunçalp

**Affiliations:** 1 Norwegian Institute of Public Health, Oslo, Norway; 2 Health Systems Research Unit, South African Medical Research Council, Cape Town, South Africa; 3 Evidence-Based Medicine Consultancy Ltd., Bath, United Kingdom; 4 Department of Reproductive Health and Research including UNDP/UNFPA/UNICEF/WHO/World Bank Special Programme of Research, Development and Research Training in Human Reproduction (HRP), World Health Organization, Geneva, Switzerland; 5 University of Central Lancashire, Preston, United Kingdom

**Keywords:** Evidence-to-decision framework, GRADE, GRADE-CERQual, Guideline development, Guideline implementation, QES, Qualitative evidence synthesis/syntheses, Qualitative methods, Qualitative systematic review, WHO guidelines

## Abstract

**Background:**

This is the third in a series of three papers describing the use of qualitative evidence syntheses (QES) to inform the development of clinical and health systems guidelines. WHO has recognised the need to improve its guideline methodology to ensure that decision-making processes are transparent and evidence based, and that the resulting recommendations are relevant and applicable to end users. In addition to the standard data on effectiveness, WHO guidelines increasingly use evidence derived from QES to provide information on acceptability and feasibility and to develop important implementation considerations.

**Methods:**

WHO convened a group drawn from the technical teams involved in formulating recent (2010–2018) guidelines employing QES. Using a pragmatic and iterative approach that included feedback from WHO staff and other stakeholders, the group reflected on, discussed and identified key methods and research implications from designing QES and using the resulting findings in guideline development. As members of WHO guideline technical teams, our aim in this paper is to explore how we have used findings from QES to develop implementation considerations for these guidelines.

**Results:**

For each guideline, in addition to using systematic reviews of effectiveness, the technical teams used QES to gather evidence of the acceptability and feasibility of interventions and, in some cases, equity issues and the value people place on different outcomes. This evidence was synthesised using standardised processes. The teams then used the QES to identify implementation considerations combined with other sources of information and input from experts.

**Conclusions:**

QES were useful sources of information for implementation considerations. However, several issues for further development remain, including whether researchers should use existing health systems frameworks when developing implementation considerations; whether researchers should take confidence in the evidence into account when developing implementation considerations; whether qualitative evidence that reveals implementation challenges should lead guideline panels to make conditional recommendations or only point to implementation considerations; and whether guideline users find it helpful to have challenges pointed out to them or whether they also need solutions. Finally, we need to explore how QES findings can be incorporated into derivative products to aid implementation.

## Background

One of the main activities of WHO is to support decision-makers globally by producing guidance about best healthcare practice [1]. The development of a WHO guideline is a lengthy process involving a number of stages, including agreeing on the topic, identifying and assessing the best available evidence, and reaching consensus about recommendations. Throughout this process, the developers of the guideline are expected to ensure that the topic, the evidence used, and the recommendations that emerge are as relevant as possible to guideline implementers in the future. WHO therefore involves not only staff members and external methodologists in these processes, but also content experts and end-users such as programme managers and health professionals, as it is they who will ultimately adopt, adapt and implement these recommendations [2].

While WHO guidelines aim to be as relevant as possible to implementers, they are not intended to include specific plans for implementation at a national or subnational level [2]. Global guidelines need to be adapted to local circumstances before they can be implemented, and local implementers are far better placed to develop these plans [3]. Nevertheless, the global evidence that is gathered to inform WHO recommendations represents a source of information that can also have value for local use. This is particularly the case for WHO guideline processes that include qualitative evidence. This type of evidence often describes how healthcare services are operationalised, viewed and experienced by stakeholders across many settings. Qualitative findings can offer future implementers an opportunity to learn from the experiences of others.

### Evidence-to-decision (EtD) frameworks and expanding the evidence base in WHO guidelines

WHO has traditionally focused on the effectiveness of an intervention in guideline processes, but this approach has begun to change in recent years. This is at least partly driven by the WHO’s increased use of EtD frameworks [2] such as the GRADE (Grading of Recommendations, Assessment, Development and Evaluation) EtD framework [4]. These frameworks help guideline panels and other decision-makers think more systematically through additional factors before reaching their final recommendation. These factors include the cost of the intervention, its acceptability and feasibility, its impact on equity, and the value people place on different outcomes [4].

Systematic reviews of randomised trials are commonly acknowledged as the best source of evidence when assessing intervention effectiveness. Systematic reviews of qualitative research, also known as qualitative evidence syntheses (QES), are, however, better suited for questions of acceptability and feasibility. The WHO guidelines on task shifting for maternal and newborn health [5] were the first to include QES in the guideline process [6]. They used QES to explore people’s experiences of lay health worker programmes [7] and nurse-doctor substitution [8]. The reviews gave valuable information about how acceptable these programmes were to service users and health workers, shedding light on important feasibility and equity issues as well as enabling the development of considerations for the people who implement the recommendations.

### Implementation considerations in EtD frameworks

Although the global guidelines of WHO generally do not include implementation plans, EtD frameworks encourage guideline panels to list broader implementation considerations [9]. These are not intended to serve as technical manuals or detailed implementation plans. Instead, they are probes, prompts, suggestions or requirements that implementers should consider when developing their local plans.

Some guidance is provided about topics that guideline teams and panel members could consider when developing implementation considerations [10]. However, there is less guidance about where this information should come from. Therefore, during our work on the technical teams of several WHO guidelines, we explored how we can use evidence from QES, combined with insights from the guideline panel, as a main source of information for the implementation considerations developed as a part of the guideline.

**Fig. 1:**
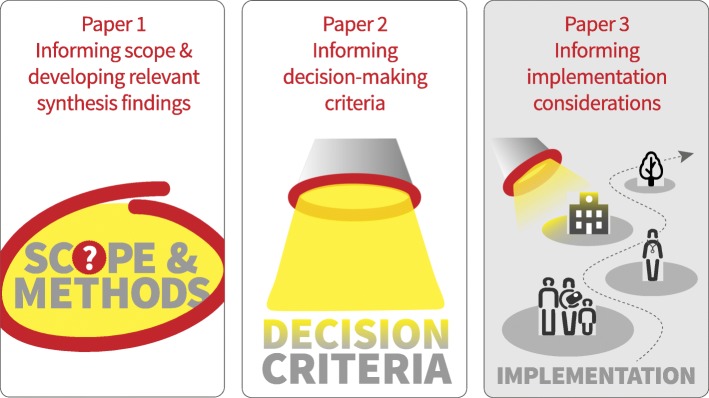
Overview of the ‘Qualitative evidence synthesis in guidelines’ series of papers

This is the third paper in a series examining the use of QES in developing clinical and health systems guidelines (Fig. 1). The focus of the first paper is on how to adapt QES methods for the guideline context [11], whereas the second focuses on how to use findings from QES to populate EtD frameworks [12]. In this final paper, we describe how, as members of guideline technical teams, we have used the findings from QES to develop implementation considerations for WHO guidelines. We include lessons learnt and point to areas where there is a need for more research and development.

## Methods

The experiences, guidance and data presented in this series of papers are the result of processes that have evolved over a decade of engagement with qualitative research in the context of developing healthcare guidelines at WHO. To develop the ideas described in the series, we used a pragmatic and iterative approach that included the following steps:

1. The WHO convened a core team of authors who had been involved in WHO guideline technical teams since 2010 and in developing QES to support these guidelines.
2. The core author team reflected on the guideline development processes in which we had been involved (see list below), focusing on the role of QES findings in these processes. We also received informal feedback on these processes from other WHO staff involved in guideline development, and from participants in several guideline training workshops at WHO. These reflections and feedback led us to identify three key areas, each of which became a focus for one of the papers in the series.
3. The lead author for each paper then drafted an outline for their paper, and these were discussed during a 4-day author workshop. In the workshop, authors discussed the most important factors in the use of qualitative evidence in this context to date and agreed on what worked and what could be improved in the future. The outlines were then developed into full papers, using an iterative process of sequential writing and discussion. We also identified relevant examples from the guidelines in which we had been involved. The core authors then reviewed the draft to clarify the ideas and processes described and to add further examples where needed.
4. We then circulated the draft papers to key stakeholders to obtain their feedback on the ideas and processes described. These stakeholders included members of WHO guideline panels (sometimes called Guideline Development Groups), methodologists, guideline commissioners and implementation experts (see *Acknowledgements*).

We selected examples from the following WHO guidelines, in the compilation of which members of the core author team had been involved:

1. Optimising health worker roles for maternal and newborn health through task-shifting (2012) [5].
2. Expanding health worker roles to help improve access to safe abortion and post-abortion care (2015) [13].
3. WHO recommendations on antenatal care for a positive pregnancy experience (2016) [14].
4. WHO recommendations on intrapartum care for a positive childbirth experience (2018) [15].
5. Guidance on communication interventions to inform and educate caregivers on routine childhood vaccination in the African Region (under review).
6. WHO recommendations on digital interventions for health systems strengthening [16].

All of these guidelines were health systems focused or had a health systems component, and all used the GRADE EtD frameworks [9]. The latter are documents with a common structure that includes a question, an assessment of the evidence that addresses the question, and a conclusion, all of which facilitate explicit and transparent decision-making [4]. The examples we present here were selected in order to highlight how the technical teams used qualitative evidence in the guideline processes and the strategies that they used to interpret and use this evidence when developing implementation considerations.

## Results

### Moving from qualitative evidence to implementation considerations

#### What was the work process?

**Fig. 2:**
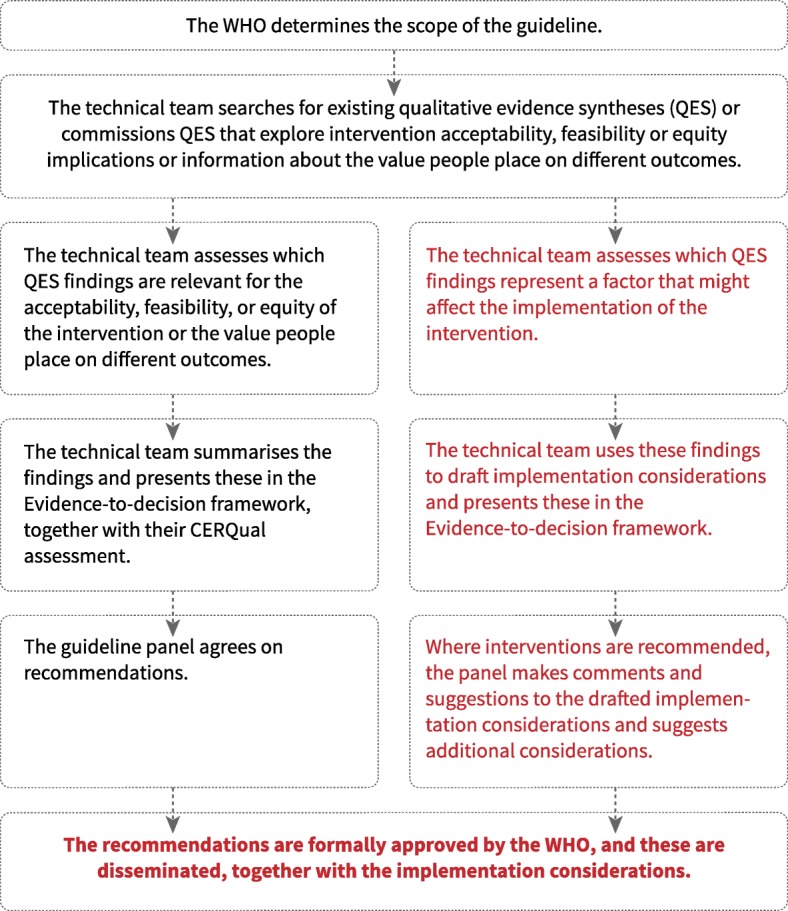
Moving from qualitative evidence to implementation considerations – the work process

For each guideline, a number of processes took place before the technical teams could begin developing the implementation considerations (Fig. 2).

First, WHO, with input from external stakeholders, agreed on the scope of the guideline and the questions it would cover. The technical team then created EtD frameworks for each of the guideline’s questions. To populate each framework’s acceptability, feasibility and equity sections, the team searched for existing QES that explored how stakeholders viewed and experienced the interventions in question. In at least two guidelines [14, 15], we also did this for the ‘values’ criterion. However, in most cases, the WHO had to commission the syntheses needed to populate the EtD frameworks, as existing QES were often lacking, out-of-date or failed in other ways to meet the needs of this guideline. Most of these commissioned QES were registered as Cochrane reviews and therefore met minimum quality standards set by Cochrane for these types of reviews [17]. For each commissioned QES, the technical team asked authors to assess how much confidence they had in each of the findings, using the GRADE-CERQual (Confidence in the Evidence from Reviews of Qualitative research) approach [18].

As soon as draft versions of the QES were ready, the technical teams extracted those findings that they considered to be particularly relevant for the acceptability, feasibility or equity of one or more of the interventions. The teams then summarised the findings, often in dialogue with the review author team, and added these to the relevant frameworks.

The approach the technical teams used when populating the feasibility, acceptability and equity criteria followed the same process used for populating the section on intervention effectiveness. In each case, the team presented findings from each review separately, made it clear that these findings were based on research evidence, and presented each finding together with its GRADE or GRADE-CERQual assessment.

Once these sections of the framework were complete, the technical team turned its attention to how the QES findings could be translated into implementation considerations. Here, the team used an approach that allowed for more interpretation of the evidence and for the inclusion of other sources of information. The team went through each QES finding and assessed the extent to which it represented a factor that might affect the implementation of the intervention in question. These findings were then used to develop a list of implementation considerations. In some cases, the team combined this information with other sources of information, including research-based or non-research-based information from the EtD frameworks, information from external sources and input from invited stakeholders. In most cases, this additional information added little to the main text about implementation considerations and the reliability of this additional information was not formally assessed.

The technical team included the first drafts of the implementation considerations in the relevant EtD frameworks. These frameworks were the main documents used by the guideline panel at the final guideline meeting, when panel members were asked to assess the evidence regarding effectiveness, resource use, acceptability, feasibility and equity before choosing to (1) recommend the intervention, (2) give a conditional or context-specific recommendations, or (3) recommend against the intervention. Where the panel chose option (3), i.e. to recommend against the intervention, the technical team removed the drafted implementation considerations as these were no longer relevant. Where the panel chose options (1) or (2), they were asked to comment on the drafted implementation considerations and to suggest additional implementation considerations.

After this meeting, the technical team edited the implementation considerations in response to comments and suggestions and presented these in the final versions of the frameworks. Once the recommendations had been formally approved by WHO, they were disseminated together with the implementation considerations.

#### When does the qualitative evidence lead to a conditional recommendation and when does it become an implementation consideration?

In many cases, the evidence from the QES pointed to problems with the acceptability or feasibility of the intervention or problems tied to equity. In some cases, this evidence led the panel to give a conditional or context-specific recommendation. This type of recommendation generally includes a description of the conditions under which the end-user should or should not implement the recommendation [2]. In other cases, the panel decided to recommend the intervention without conditions, and to deal with these concerns as implementation considerations only. For instance, in the antenatal care guideline [14], the panel was asked to decide whether to recommend a midwife-led continuity-of-care model. One QES finding showed that women appreciated being seen by the same healthcare professional at each appointment because this gave them the opportunity to build caring, trusting relationships with these providers [19]. However, additional information from the same QES and input from the guideline panel suggested that the lack of trained midwives and the potential for burnout of health professionals from work overload often reduced the feasibility of this model of care. Taking these feasibility issues into account, the panel agreed to make a context-specific recommendation, recommending this model of care only in settings with well-functioning midwifery programmes. In addition, under ‘Implementation considerations’, the requirements and conditions that would need to be in place for this model of care to be implemented were reiterated (Example 1, Table 1).

**Table 1:** Examples of implementation considerations influenced by qualitative findings

Example	Qualitative evidence synthesis (QES) finding/s		Other considerations/sources of information	Implementation considerations^a^
*1*	RECOMMENDATION: Midwife-led continuity-of-care (MLCC) models, in which a known midwife or small group of known midwives supports a woman throughout the antenatal, intrapartum and postnatal continuum, are recommended for pregnant women in settings with well-functioning midwifery programmes [14]			
	From QES about factors affecting uptake and quality of routine antenatal care services [19]: Women appreciated being seen by the same healthcare professional at each appointment (including pre and post-natal) primarily because this gave them the opportunity to build caring, trusting relationships with healthcare providers (high confidence in the evidence).		Additional information from the same QES and input from the guideline panel suggested that the lack of trained midwives and the potential for health professional burnout from work overload often reduced the feasibility of this model of care.	Need to know: what model of care is currently being used; whether there are sufficient numbers of trained midwives; and whether resources are available or can be shifted to facilitate this model. Need to have: a well-functioning midwifery programme. Need to do: consult all relevant stakeholders, including human resource departments and professional bodies; assess the need for additional training in MLCC; ensure that there is a well-functioning referral system in place; and monitor midwife workload and burnout. Need to consider: strategies to scale-up the quality and number of practising midwives; ways of providing continuity-of-care through other care providers, e.g. lay health workers (LHWs); and whether a caseload or team MLCC model is more appropriate.
2	RECOMMENDATION: Epidural analgesia is recommended for healthy pregnant women requesting pain relief during labour, depending on a woman’s preferences [15]			
	From QES on experiences of labour and childbirth [20]: ... there were mixed views of epidural analgesia usage. Views were influenced by the availability of epidural analgesia, and by accounts of others (moderate confidence in the evidence). Some women expressed an a priori desire for an epidural analgesia to help with a pain-free labour, to alleviate a fear of pain and/or to remain in control during labour (moderate confidence in the evidence), while others requested an epidural as a last resort, when the level of pain and/or sense of control over the labour was overwhelming and unmanageable (low confidence in the evidence). A perceived lack of effectiveness of epidural analgesia use reported by women in some studies was partly attributed to late administration (low confidence in the evidence), suggesting that there might be logistical issues in implementing this pain relief method.		All of the findings on epidural use came from high-income country settings where epidural analgesia is widely available. In lower-resource settings, where it is not so widely used, there are likely to be financial implications as well as additional training considerations, which may negatively impact on the feasibility of implementing this intervention. Evidence on the resources required for this intervention were also considered by the panel.	Policy-makers need to determine which pain relief measures are most feasible and acceptable in their settings. Facilities offering epidural analgesia need to have staff with the appropriate specialist skills (anaesthetists, obstetricians), as well as equipment and systems in place to monitor, detect and manage any undesirable effects of the procedure during and after labour to ensure the safety of mother and baby. Epidural analgesia should not be introduced in settings where these resources are not consistently available.
*3*	General implementation considerations from “Guidance on communication interventions to inform and educate caregivers on routine childhood vaccination in the African Region” (under review)			
	From QES about perceptions of childhood vaccination information [21]: Parents generally find the amount of vaccination information they are currently receiving to be inadequate (high confidence). They find some of this information to be too general and want specific information on: the benefits of different vaccines as well as possible side effects • how vaccines are made and how they work • the diseases the vaccines are designed to prevent • the differences between different vaccines • the vaccination schedule and what to expect at the vaccine appointment (high confidence)			Different people are likely to have different information needs. However, all people should have easy access to information about the benefits of different vaccines as well as possible side effects; how vaccines are made and how they work; the diseases the vaccines are designed to prevent; the differences between different vaccines; the vaccination schedule; and what to expect at the vaccine appointment.
*4*	RECOMMENDATION: We recommend the use of LHWs to provide continuous support during labour, in the presence of a skilled birth attendant. However, appropriate attention must be paid to the acceptability of the intervention to other healthcare providers [5]			
	From QES about the implementation of lay health worker programmes [7]: Activities that demand that the LHW is present at specific times, for instance during labour and birth, lead to irregular and unpredictable working conditions. This may have direct implications for LHWs’ expectations regarding incentives (low certainty evidence). LHWs may also be concerned about personal safety when working in the community and some LHWs were reluctant to visit clients at night because of safety issues (moderate certainty evidence).			Systems need to be in place to support LHWs who may need to travel at night in order to assist during labour and delivery.
5	General implementation considerations from “Guidance on communication interventions to inform and educate caregivers on routine childhood vaccination in the African Region)” (under review)			
	From QES about perceptions of childhood vaccination information [21]: People’s trust in and relationship with the information source: parents find it difficult to know which vaccination information sources to trust and to find a source that they perceive as impartial or balanced. They feel that the information they receive is biased towards vaccination (high confidence). Some parents distrust or lack confidence in information sources linked to the government. They consider these sources to be biased, to be withholding information, or to be motivated by financial gain (moderate confidence). Health workers are an important source of vaccination information for many parents (high confidence). Some parents vaccinate their children because they perceive their health workers as trustworthy and helpful (moderate confidence). However, some parents feel rushed and intimidated into vaccinating, or judged and pressured by health workers (moderate confidence). Some parents, especially vaccine-hesitant parents, also question whether health workers’ motives are tied to financial gain (moderate confidence). Parents who have less trust in their health worker or the information they provide, may search for more information from other sources (low confidence). Politicians’ opinions and actions regarding personal vaccination choices may also influence parents’ perceptions of vaccination (low confidence).		Knowledge and expertise from within the guideline panel and the technical team.	Community members should feel confident that health workers and others communicating vaccination information are driven primarily by the best interests of the child and are trustworthy, balanced and impartial. Consider involving LHWs or other members of the community, including religious or political leaders, if these are viewed by communities as trustworthy sources.
*6*	RECOMMENDATION: Respectful maternity care (RMC), which refers to care organised for and provided to all women in a manner that maintains their dignity, privacy and confidentiality, ensures freedom from harm and mistreatment, and enables informed choice and continuous support during labour and childbirth, is recommended [15]			
	From QES on RMC [22]: Findings from a qualitative review indicate that women appreciate RMC across countries and settings (high confidence in the evidence). Stakeholders (including women, providers and administrators) emphasised the theoretical importance of providing and ensuring RMC for all women. Review findings also suggest that efforts to address or improve RMC may be acceptable to healthcare providers (high confidence in the evidence). However, in environments where resources are limited, healthcare providers believe that RMC could increase their workload and could reduce their ability to provide quality care to all women.		The WHO’s normative position and the UNHRC’s report on a rights-based approach to maternal health strongly influenced the guideline panel and technical team [23]. Individual studies suggested that mistreatment of women during childbirth is often due to existing social norms and, in some settings, it may be regarded by healthcare providers and other stakeholders as acceptable [22, 24].	Mechanisms should be put in place to ensure that all women, and particularly those from disadvantaged backgrounds, are made aware of (1) their right to RMC and (2) the existence of a mechanism for raising and addressing complaints (e.g. an audit and feedback mechanism that integrates women’s complaints and ensures that responses are provided)

^a^In most instances, these implementation considerations are excerpts from the respective guidelines and readers are encouraged to refer to the respective guidelines for other related implementation considerations

In another example from the digital health guidelines, due to the potential for harm introduced by disclosing health information, ensuring the confidentiality of a communication about sensitive health topics was perceived to be a prerequisite for executing the recommendation on whether to transmit health information via mobile devices. This was therefore formulated as a conditional recommendation. The evidence on other factors to enhance the acceptability of the intervention, such as allowing users to unsubscribe or to determine the frequency of communication content they received, was translated into implementation considerations.

For other recommendations, the guideline panel decided to recommend the intervention without conditions, and to address these types of concerns only in the implementation considerations section. For instance, feasibility concerns about network connectivity and access to electricity highlighted by the QES in the digital health guidelines [25] were dealt with as implementation considerations and did not lead to conditional recommendations. Similarly, in the intrapartum care guideline, acceptability and feasibility concerns were addressed in the implementation considerations on epidural analgesia, which was unconditionally recommended by the panel (Example 2, Table 1).

##### Lessons learnt

It was not always clear why some concerns identified in the qualitative evidence led the panel to make a conditional recommendation while others were only dealt with as implementation considerations. In some cases, this may have been tied to the panel’s perceptions of how serious these concerns were and if they were prerequisites to establishing the recommendation. In other cases, the panel’s decision may have been influenced by other factors, such as their assessment of the intervention’s effectiveness or its impact on equity. The panel also tended to be cognisant of not placing too many conditions on a recommendation, as this can make it difficult for stakeholders to determine whether it is appropriate to implement the intervention or not.

WHO guidelines have previously been criticised for making ‘strong recommendations’ despite only low or very low confidence evidence of effectiveness [26, 27]. Panels are now increasingly expected to consider evidence about factors such as acceptability, feasibility and equity, in addition to evidence about effectiveness. Future research should explore if and when problems about issues that have been highlighted by qualitative evidence should lead to conditional recommendations or only to implementation considerations.

#### How was the evidence transformed?

The process from QES finding to implementation consideration involved varying degrees of transformation. In some cases, it involved little editing of the finding, yet, in others, the information that emerged from the finding was combined with other evidence or information.

When a QES finding pointed to elements of an intervention that stakeholders, such as clients or health workers, particularly liked or wanted, the technical team often rephrased the finding as an implementation consideration with little editing. For instance, in the guidelines on communication interventions for childhood vaccination, the panel decided to recommend the use of face-to-face communication interventions directed at parents or caregivers (Meeting of the WHO African Regional Office (WHO-AFRO), 2018 [11]). One of the supporting QES showed that parents generally wanted more information than they were currently receiving and described the types of information they would like to receive [21]. In this case, the team simply listed the types of information that implementers should consider offering to parents under ‘Implementation considerations’ (Example 3, Table 1).

Similarly, when a QES finding pointed to elements of an intervention that stakeholders found less acceptable or challenging to implement, the team often rephrased the finding as an implementation consideration with little editing. For instance, in the guidelines on task-shifting for maternal and newborn health [5], the panel decided to recommend the use of lay health workers to provide continuous support to women during labour. One of the QES showed that some lay health workers were concerned about personal safety when working in the community and were reluctant to visit clients at night because of safety issues [7]. Here, under ‘Implementation considerations’, the technical team raised awareness of these problems and the need for implementers to address them (Example 3, Table 1).

In other cases, the team attempted to offer suggestions or solutions to problems identified in the QES, often drawing on other sources of information. For instance, in the guidelines on communication interventions for childhood vaccination (under review), the QES showed that people found it difficult to know which vaccination information sources to trust, and that some suspected information sources as being motivated by financial gain. In the implementation considerations, the technical team highlighted this issue, but also suggested that implementers should consider involving community-based health workers or other members of the community, including religious or political leaders, as people viewed these individuals as trustworthy (Example 4, Table 1). When making these suggestions, the team drew on other QES findings, but also on knowledge and expertise of the panel and the technical team.

##### Lessons learnt

It is possible that simply drawing attention to what people want or need or to existing problems may be helpful to implementers. However, these types of implementation considerations may be less useful than those that also suggest solutions. Future research should explore the extent to which implementers experience the identification of problems alone as useful. The usefulness of suggested solutions may also depend on how evidence based and generalisable they are. Some of the solutions suggested were drawn from the QES and were gathered in a systematic and transparent way. Others were less systematically developed but were drawn from the knowledge of individuals such as members of the technical team or the guideline panel. The extent to which this is a problem is uncertain but leads us to the next issue, namely, the extent to which implementation considerations should reflect our confidence in the underlying qualitative evidence.

#### What factors influenced how the implementation considerations were phrased?

The team’s choice of language and degree of caution when formulating implementation considerations was sometimes influenced by our level of confidence in the underlying qualitative evidence as assessed using GRADE-CERQual. For instance, the suggestion that implementers consider the use of political leaders as one way of addressing people’s trust issues surrounding sources of information on vaccination was based on a qualitative finding assessed as being of ‘low confidence’, in addition to expert opinion (Example 4, Table 1). In these cases, the technical team often tried to avoid terms such as “*implementers should …*”, and used more cautious language, e.g. “*…consider involving … members of the community, including religious or political leaders…*”. However, the different teams did not use this approach consistently.

The team’s choice of language and degree of caution exercised were also influenced by the extent to which the findings from the qualitative evidence reflected the normative positions and principles held by WHO. For instance, a QES developed for the intrapartum care guideline [16] indicated that women from all countries and settings want and appreciate respectful maternity care (RMC) [22]. The principle of RMC is emphasised by WHO and underpinned by a human rights-based approach [23]. As disrespectful care remains prevalent in all sorts of maternity contexts, particularly for vulnerable or marginalised women, the technical team used the ‘Implementation considerations’ section to emphasise to stakeholders that mechanisms need to be put in place to ensure that all women are made aware of their right to RMC, and how to raise complaints should they not receive it (Example 6, Table 1). In this case, when formulating the implementation consideration, the team deliberately used the word ‘should’ to stress this normative standpoint. Similarly, in the QES on parents’ perceptions of childhood vaccination information [21] developed for the guideline on the same topic (under review), parents called for far more information about vaccination. As information is considered by WHO to be a universal right, the language used when formulating this implementation consideration was deliberately emphatic: “*Different people are likely to have different information needs. However, all people should have easy access to information about …*” [16, 23] (Example 2, Table 1). However, again, the different guideline teams did not use this approach consistently.

##### Lessons learnt

Ideally, the language used when presenting implementation considerations should reflect the level of confidence in the evidence on which it is based. In many cases, the underlying evidence might be downgraded to low or very low confidence, for instance, because of concerns about its relevance or because of serious methodological limitations. In these cases, technical teams should consider formulating implementation considerations more cautiously, i.e. as questions, prompts or suggestions. Alternatively, they should consider basing the implementation considerations primarily on those qualitative findings that have been assessed as being of moderate or high confidence.

One circumstance that may override the confidence in the QES evidence is the presence of overarching ideals and principles held by the body producing the guideline (in this case, WHO), including principles associated with human rights). In such cases, the team has often phrased the implementation considerations in relatively strong terms that are closer to recommendations than prompts or suggestions, and this has sometimes been done irrespective of the level of confidence in the evidence. However, strong terms, including the use of the word ‘should’, need to be carefully considered. Therefore, when using this approach, guideline commissioners, technical teams and panels should consider adopting a more reflexive and transparent approach early on in the guideline process where they explicitly identify the overarching principles, including normative values, driving the guideline. To achieve this, it may be helpful to borrow from reflexive exercises recommended within qualitative research practice, as described in Paper 1 of this series [11], and to explore how these can be carried out at different stages of the guideline process.

#### Can existing health systems frameworks be used to organise implementation considerations and identify gaps?

For some of the guidelines, the technical team used existing health systems frameworks to help organise the implementation considerations into meaningful groups. For instance, the team used the WHO Building Blocks [28] and the SURE Framework [29] to help organise implementation considerations in the guidelines on task-shifting for maternal and newborn health [5], and the WHO/ITU National eHealth Strategy Toolkit [30] in the digital health guidelines [16, 31].

While these frameworks helped us group implementation considerations, they also highlighted gaps in the qualitative evidence. The qualitative evidence was often a good source of data for implementation considerations at the level of the facility, the community or the individual, but it was less rich for factors associated with higher levels of the health system. For instance, a QES [25] used in the digital health guidelines [16] described different challenges to the implementation of these interventions, including health worker motivation, workload, training and supervision issues; the impact on health worker relationships with clients, the community and other health workers; and access to supplies, electricity and network connectivity. Similarly, a QES used in the guidelines on childhood vaccination communication described parents’ needs and challenges regarding information content, format, source and timing, as well as issues tied to trust and decision-making [21]. However, financing, legal and political factors tended to be poorly represented in the data.

In some cases, the technical team addressed this problem by drawing on information from other sources to develop implementation considerations. For instance, in the guidelines on task-shifting for maternal and newborn health [5] and for abortion care [13], the team carried out case study syntheses to identify these more ‘upstream’ system-level factors in selected countries [32, 33]. Here, a variety of sources were used, including published research, programme reports, and interviews with key informants. However, in other cases, these domains remained underrepresented in the frameworks.

##### Lessons learnt

The teams did not use health systems frameworks to organise implementation considerations in all the guidelines, and where the teams did use them, this was after the implementation considerations had been developed. GRADE recommends the use of these types of frameworks, such as the TICD (Tailored Interventions for Chronic Diseases) Checklist [10], not only to organise implementation considerations but also to identify them. In future guideline processes, technical teams should consider more active use of such frameworks at an earlier stage of the process.

As mentioned, these frameworks also helped us to identify where there were gaps in the evidence. Although circumstances at higher levels of the health system are likely to be important for the successful implementation of most healthcare interventions, qualitative evidence tended to focus on the level of the individual and on lower levels of the health system. This was probably partly due to how the QES were framed. It likely also reflects traditions within qualitative research environments. However, it does not reflect any inherent limitations of this research methodology. Authors of primary qualitative research should be encouraged to explore views, experiences and processes at higher levels of the system to a greater extent. Meanwhile, future technical teams should consider the scope of commissioned QES and also consider how they can gather direct input from higher level stakeholders, for instance, by greater use of key informant interviews.

#### When are overall implementation considerations made versus recommendation-specific implementation considerations?

In many cases, the QES findings were only relevant for specific recommendations or groups of recommendations. However, some QES findings raised issues that were relevant across the whole scope of the guideline and highlighted how broader health barriers could potentially prevent the successful implementation of all of the included interventions. For instance, in the WHO guideline on task-shifting for maternal and newborn health [5], the QES pointed to inadequate health worker training among all relevant health worker cadres and for diverse tasks. In other cases, QES highlighted even broader barriers such as infrastructure problems beyond the health system. For instance, in the digital health guidelines [16], the QES pointed to widespread problems with network connectivity and access to electricity that could ultimately prevent the implementation of all the included interventions. The QES also identified healthcare users’ needs and preferences that cut across the individual guideline recommendations. For instance, in the intrapartum care guideline [15], the QES showed that most women preferred to have a normal birth without unnecessary interventions, although they did appreciate that medical interventions are sometimes necessary [20]. In addition to recommendation-specific implementation considerations, the technical team therefore developed an overview of cross-cutting implementation considerations applicable to all the recommendations. The team presented this information outside of the EtD framework format, as this format currently does not allow for this type of cross-cutting information.

##### Lessons learnt

The primary aim of the technical team was to use the QES findings to develop implementation considerations that were relevant to specific recommendations in the guideline. These recommendations are often relatively narrow, usually following the PICO (Population, Intervention, Comparison, Outcome) approach used for research on effectiveness. Qualitative research, on the other hand, is designed to be more explorative, and focuses on people’s own experiences and categorisations. In practice, this means that qualitative data are rarely confined to the boundaries of ‘PICO’, and are generally broader in scope. This has practical implications when using qualitative evidence, not only when developing implementation considerations, but also when gathering information about acceptability, feasibility and equity, as it may be difficult to categorise findings as belonging to specific EtD frameworks. While the technical team solved this problem by developing cross-cutting presentations of implementation considerations, future research should explore how the broader issues identified through qualitative evidence can be acknowledged and incorporated into the design of the EtD frameworks.

## Discussion

In a series of WHO guideline processes where we worked on the technical teams, we used QES as our main source of information when developing implementation considerations. As we have gained experience, our processes for doing so have been fine-tuned and we have learnt a number of lessons, summarised in Box 1.

Nevertheless, the transition from qualitative evidence to implementation considerations has highlighted a number of issues that deserve further discussion, including whether researchers should use existing health systems frameworks when developing implementation considerations; whether researchers should take confidence in the underlying evidence into account when developing implementation considerations; whether qualitative evidence that reveals implementation challenges should lead guideline panels to make conditional recommendations or only point to implementation considerations; and whether guideline users find it helpful to have challenges pointed out to them or whether they also need solutions to be suggested. Finally, we need to explore how intervention-specific EtD frameworks can be adapted to allow for broader, cross-cutting issues identified through qualitative research.

Despite these issues, our experiences suggest that qualitative evidence represents a useful source of information for future implementers. By systematically gathering global evidence about people’s experiences of clinical and health systems interventions, guidelines can offer useful information that local implementers can learn from. However, these implementers still need to adapt the guidelines to their local settings. We are currently exploring different approaches to assist them in doing so, including how logic models based on qualitative evidence and local data can be used to inform derivative products (e.g. manuals, toolkits) for guideline adaptation and implementation.

## Conclusions

As members of the guideline technical teams, we experienced QES as a useful source of information when developing implementation considerations. However, questions still remain about how researchers should prepare and present implementation considerations, how guideline panels should respond to implementation challenges when reaching recommendations, and how we can prepare information about implementation that guideline users find useful. The use of derivative products to further assist these end users also needs further exploration.

Box 1 Implications of lessons learnt Implications for practice

- Technical teams should consider using health systems frameworks at an early stage of the process, not only to organise implementation considerations but also to identify them.
- To increase access to qualitative evidence about higher levels of the health system, technical teams should consider the scope of commissioned QES and should also consider how they can gather direct input from higher-level stakeholders, for instance, through an increased use of key informant interviews or surveys.
- Implementation considerations may also be influenced by overarching ideals and principles held by the guideline-producing body, including principles tied to human rights. Guideline commissioners, technical teams and panels should consider adopting a more reflexive and transparent approach early on in the guideline process, where they identify these overarching principles.
- Where confidence in the qualitative evidence is low or very low, technical teams should consider formulating implementation considerations based on this evidence cautiously, for instance, as questions, prompts or suggestions. Alternatively, they should consider basing the implementation considerations primarily on those qualitative findings that have been assessed as being of moderate or high confidence.

Implications for research

- Authors of primary qualitative research should be encouraged to explore views, experiences and processes at higher levels of the system to a greater extent.
- Future methodological research should explore:
- How broader issues identified through qualitative evidence can be acknowledged and incorporated into the design of the EtD frameworks.
- If and when problems tied to values, acceptability, feasibility or equity that have been highlighted by the qualitative evidence should lead to conditional recommendations or to implementation considerations.
- Whether it is helpful to draw implementers’ attention to the needs and preferences of target audiences and to possible implementation problems, or if information about solutions is also necessary.

## Abbreviations

CERQual: Confidence in the Evidence from Reviews of Qualitative research
EtD: evidence-to-decision (frameworks)
GRADE: Grading of Recommendations, Assessment, Development and Evaluation
QES: qualitative evidence synthesis/syntheses
RMC: respectful maternity care
WHO-AFRO: WHO African Regional Office

## References

[1] World Health Organization. Constitution of the World Health Organization. http://apps.who.int/gb/bd/PDF/bd47/EN/constitution-en.pdf?ua=1. Accessed 21 Jun 2019.

[2] World Health Organization (2014). WHO Handbook for Guideline Development.

[3] Wang Z, Norris SL, Bero L (2018). The advantages and limitations of guideline adaptation frameworks. Implement Sci.

[4] Alonso-Coello P, Schünemann HJ, Moberg J, Brignardello-Petersen R, Akl EA, Davoli M (2016). GRADE Evidence to Decision (EtD) frameworks: a systematic and transparent approach to making well informed healthcare choices. 1: Introduction. BMJ.

[5] World Health Organization. WHO Recommendations: Optimizing Health Worker Roles to Improve Access to Key Maternal and Newborn Health Interventions through Task Shifting. 2012. http://www.who.int/iris/handle/10665/77764. Accessed 21 Jun 2019. 23844452

[6] Glenton C, Lewin S, Gülmezoglu AM (2016). Expanding the evidence base for global recommendations on health systems: strengths and challenges of the OptimizeMNH guidance process. Implement Sci.

[7] Glenton C, Colvin CJ, Carlsen B, Swartz A, Lewin S, Noyes J (2013). Barriers and facilitators to the implementation of lay health worker programmes to improve access to maternal and child health: qualitative evidence synthesis. Cochrane Database Syst Rev.

[8] Rashidian A, Shakibazadeh E, Karimi- Shahanjarini A, Glenton C, Noyes J, Lewin S (2013). Barriers and facilitators to the implementation of doctor-nurse substitution strategies in primary care: qualitative evidence synthesis (protocol). Cochrane Database Syst Rev.

[9] Moberg J, Oxman AD, Rosenbaum S, Schünemann HJ, Guyatt G, Flottorp S (2018). The GRADE Evidence to Decision (EtD) framework for health system and public health decisions. Health Res Policy Syst.

[10] Flottorp SA, Oxman AD, Krause J, Musila NR, Wensing M, Godycki-Cwirko M (2013). A checklist for identifying determinants of practice: a systematic review and synthesis of frameworks and taxonomies of factors that prevent or enable improvements in healthcare professional practice. Implement Sci.

[11] Downe S, Finlayson K, Lawrie TA, Lewin S, Glenton C, Rosenbaum S, et al. Qualitative evidence synthesis (QES) for guidelines: Paper 1. Using qualitative evidence synthesis to inform guideline scope and develop qualitative findings statements. Health Res Policy Syst. 2019;17. 10.1186/s12961-019-0467-5. 10.1186/s12961-019-0467-5 PMC6686511 31391057

[12] Lewin S, Glenton C, Lawrie TA, Downe S, Finlayson K, Rosenbaum S, et al. Qualitative evidence synthesis (QES) for guidelines: Paper 2. Using qualitative evidence synthesis findings to inform evidence-to-decision frameworks and recommendations. Health Res Policy Syst. 2019;17. 10.1186/s12961-019-0468-4. 10.1186/s12961-019-0468-4 PMC6686513 31391119

[13] World Health Organization (2015). Health Worker Roles in Providing Safe Abortion Care and Post-Abortion Contraception.

[14] World Health Organization (2016). WHO Recommendations on Antenatal Care for a Positive Pregnancy Experience.

[15] World Health Organization. WHO Recommendations: Intrapartum Care for a Positive Childbirth Experience. http://apps.who.int/iris/bitstream/handle/10665/260178/9789241550215-eng.pdf. Accessed 10 Apr 2018. 30070803

[16] World Health Organization. WHO guideline: Recommendations on Digital Interventions for Health Systems Strengthening. Geneva: WHO; 2019. 31162915

[17] Noyes J, Booth A, Cargo M, Flemming K, Garside R, Hannes K (2018). Cochrane Qualitative and Implementation Methods Group guidance series-paper 1: introduction. J Clin Epidemiol.

[18] Lewin S, Booth A, Glenton C, Munthe-Kaas H, Rashidian A, Wainwright M (2018). Applying GRADE-CERQual to qualitative evidence synthesis findings: introduction to the series. Implement Sci.

[19] Downe S, Finlayson K, Tunçalp Ö, Gülmezoglu AM. Provision and uptake of routine antenatal services: a qualitative evidence synthesis. Cochrane Database Syst Rev. 2019;6:CD012392. 10.1002/14651858.CD012392.pub2 PMC6564082 31194903

[20] Downe Soo, Finlayson Kenneth, Oladapo Olufemi, Bonet Mercedes, Gülmezoglu A. Metin (2018). What matters to women during childbirth: A systematic qualitative review. PLOS ONE.

[21] Ames HM, Glenton C, Lewin S (2017). Parents’ and informal caregivers’ views and experiences of communication about routine childhood vaccination: a synthesis of qualitative evidence. Cochrane Database Syst Rev.

[22] Shakibazadeh E, Namadian M, Bohren MA, Vogel JP, Rashidian A, Nogueira Pileggi V (2018). Respectful care during childbirth in health facilities globally: a qualitative evidence synthesis. BJOG.

[23] UNHRC (2012). Technical Guidance on the Application of a Human Rights-Based Approach to the Implementation of Policies and Programmes to Reduce Preventable Maternal Morbidity and Mortality: Annual Report.

[24] Bohren MA, Vogel JP, Tuncalp O, Fawole B, Titiloye MA, Olutayo AO (2016). “By slapping their laps, the patient will know that you truly care for her”. A qualitative study on social norms and acceptability of the mistreatment of women during childbirth in Abuja, Nigeria. SSM Popul Health.

[25] Odendaal W, Goudge J, Griffiths F, Tomlinson M, Leon N, Daniels K (2015). Healthcare workers’ perceptions and experiences on using mHealth technologies to deliver primary healthcare services: a qualitative evidence synthesis. Cochrane Database Syst Rev.

[26] Alexander PE, Bero L, Montori VM, Brito JP, Stoltzfus R, Djulbegovic B (2014). World Health Organization recommendations are often strong based on low confidence in effect estimates. J Clin Epidemiol.

[27] Alexander PE, Gionfriddo MR, Li SA, Bero L, Stoltzfus RJ, Neumann I (2016). A number of factors explain why WHO guideline developers make strong recommendations inconsistent with GRADE guidance. J Clin Epidemiol.

[28] De Savigny D, Adam T. Systems Thinking for Health System Strengthening. Geneva: WHO; 2009. http://apps.who.int/iris/bitstream/handle/10665/44204/9789241563895_eng.pdf. Accessed 21 Jun 2019.

[29] The SURE Collaboration. SURE Guides for Preparing and Using Evidence-Based Policy Briefs: Identifying and Addressing Barriers to Implementing Policy Options 2011. version 2.1. Updated Nov 2011. http://www.who.int/evidence/sure/guides/en/. Accessed 21 Jun 2019.

[30] World Health Organization. National eHealth Strategy Toolkit: Overview. Geneva: WHO; 2012. https://www.who.int/ehealth/publications/overview.pdf. Accessed 21 Jun 2019.

[31] World Health Organization. WHO Recommendations on Digital Interventions for Health Systems Strengthening. Geneva: WHO; 2019. 31162915

[32] Glenton C, Sorhaindo AM, Ganatra B, Lewin S (2017). Implementation considerations when expanding health worker roles to include safe abortion care: a five-country case study synthesis. BMC Public Health.

[33] Gopinathan U, Lewin S, Glenton C (2014). Implementing large-scale programmes to optimise the health workforce in low- and middle-income settings: a multicountry case study synthesis. Tropical Med Int Health.

